# Identification and Quantification of S-Sulfenylation Proteome of Mycobacterium tuberculosis under Oxidative Stress

**DOI:** 10.1128/spectrum.03386-22

**Published:** 2023-03-21

**Authors:** Yun Lu, Hongtong Chen, Penghe Wang, Jing Pang, Xi Lu, Guoqing Li, Xinxin Hu, Xiukun Wang, Xinyi Yang, Congran Li, Yu Lu, Xuefu You

**Affiliations:** a Beijing Key Laboratory of Antimicrobial Agents, Institute of Medicinal Biotechnology, Chinese Academy of Medical Sciences and Peking Union Medical College, Beijing, China; b Department of Pharmacology, Beijing Chest Hospital, Capital Medical University; Beijing Key Laboratory of Drug Resistance Tuberculosis Research, Beijing Tuberculosis and Thoracic Tumor Research Institute, Beijing, China; University of Guelph College of Biological Science

**Keywords:** *Mycobacterium tuberculosis*, S-sulfenylation, hydrogen peroxide, redox homeostasis, cysteine residue

## Abstract

The ability to maintain redox homeostasis is critical for Mycobacterium tuberculosis (*Mtb*) to survive the redox stress of the host. There are many antioxidant systems in *Mtb* to ensure its normal replication and survival in the host, and cysteine thiols are one of them. S-sulfenylation is one of the reversible modifications of cysteine thiols to resist oxidative stress. In the study, we investigated the total cysteine thiols modification and S-sulfenylation modification of *Mtb* proteome under the oxidative stress provided by hydrogen peroxide. To determine and quantify the S-sulfenylation modified proteins, high specific IodoTMT6plex reagents and high resolution mass spectrometry were used to label and quantify the peptides and proteins modified. There are significant differences for the total cysteine modification levels of 279 proteins and S-sulfenylation modification levels of 297 proteins under hydrogen peroxide stress. Functional enrichment analysis indicated that these cysteine-modified proteins were involved in the oxidation-reduction process, fatty acid biosynthetic process, stress response, protein repair, cell wall, etc. In conclusion, our study provides a view of cysteine modifications of the *Mtb* proteome under oxidative stress, revealing a series of proteins that may play a role in maintaining redox homeostasis.

**IMPORTANCE** With the continuous spread of drug-resistant tuberculosis, there is an urgent need for new antituberculosis drugs with new mechanisms. The ability of *Mtb* to resist oxidative stress is extremely important for maintaining redox homeostasis and survival in the host. The reversible modifications of cysteine residues have a dual role of protection from irreversible damage to protein functions and regulation, which plays an important role in the redox homeostasis system. Thus, to discover cysteine modification changes in the proteome level under oxidative stress is quintessential to elucidate its antioxidant mechanism. Our results provided a list of proteins involved in the antioxidant process that potentially could be considered targets for drug discovery and vaccine development. Furthermore, it is the first study to determine and quantify the S-sulfenylation-modified proteins in *Mtb*, which provided better insight into the *Mtb* response to the host oxidative defense and enable a deeper understanding of *Mtb* survival strategies.

## INTRODUCTION

Tuberculosis (TB), which is caused by Mycobacterium tuberculosis (*Mtb*), continues to be a public health threat. With the development and wide spread of drug-resistant TB, the pressure of prevention and treatment is increasing. WHO reported that an estimated 450,000 incident cases developed multidrug-resistant-TB or rifampicin-resistant-TB (MDR/RR -TB) in 2021, up 3.1% from 437,000 in 2020 (1). At present, the first-line anti-TB clinical drugs have been used for many years, and resistance to these drugs is emerging and developing (2). Thus, there is an urgent need for new anti-TB drugs with new mechanisms and structures, and one of the strategies to develop anti-TB drugs with novel mechanisms is to find new drug targets.

Redox homeostasis (RH) is the balance of oxidative and reductive reactions present in all living systems. The ability of *Mtb* to survive and replicate in macrophages indicates that it has a complex mechanism for maintaining RH and resisting redox stress *in vivo* (3). However, the whole mechanism of *Mtb* to maintain RH is still unknown. Exploring how host environmental factors affect the physiological functions of *Mtb* and how to cause changes in RH may help us get a deeper understanding of redox physiological functions of *Mtb* and be able to develop mycobacterial intervention strategies. Oxidative stress refers to the physiological process of the imbalance between oxidation and anti-oxidation, which leads to the increase of protease secretion and the production of a large number of oxidative intermediate products. In the process of infection and transmission, *Mtb* will encounter many exogenous and endogenous oxidative stress. For example, during the transmission by enclosed aerosols, *Mtb* faces high oxygen stress and nutrient deprivation, while in activated macrophages, *Mtb* faces oxidative pressure such as reactive oxygen intermediates and/or reactive oxygen intermediates (ROI/RNI) (3). Therefore, the ability to handle a large amount of oxidative stress produced by the host is essential for the survival of *Mtb* whether in the latent state or active state.

The redox centers of many proteins (such as iron-sulfur clusters, flavoprotein, etc.) are very sensitive to ROS (reactive oxygen species) and RNS (reactive oxygen species) produced by the host. ROS/RNS that cause protein oxidation include free radicals and nonradical substances. RNS refers to radicals, such as NO^•^ and NO_2_^•^, and nonradicals, such as ROONO, NO^+^, HNO_2_, etc. ROS refer to radicals, such as RO^•^, HO_2_^•^, HO^•^, and CO_2_^•−^ and nonradicals, such as hydrogen peroxide (H_2_O_2_), ROOH, etc. (4). ROS/RNS stress may lead to reversible and/or irreversible modification of sensitive proteins. Reversible modifications include S-sulfation (SSH), S-nitrosylation (SNO), S-sulfenylation (SOH), and disulfide (SSR) formation, while irreversible modifications include S-sulfinic acid (SO_2_H) and S-sulfonic acid (SO_3_H) (5). To resist oxidative stress, organisms have evolved enzymatic and nonenzymatic reaction mechanisms aimed at directly removing ROS and RNS or repairing oxidative damage. There are many antioxidant systems in *Mtb* to ensure its normal replication and survival in the host, including thioredoxin reductase, superoxide dismutase, alkyl hydroperoxidase, sulfhydryl peroxidase, etc. (6, 7), whose side chains can directly react with oxidants or oxidized cell products. Among these residues, cysteine residues are most commonly used.

The reversible modifications of cysteine residues have a dual role of protection from irreversible damage to protein functions and regulation, which plays a very important role in the RH system. For example, the thioredoxin system in *Mtb* and the mycothiol (MSH) system together regulate many important cellular processes, such as antioxidant pathways, DNA and protein repair enzyme activities, and the activation of redox-sensitive transcription factors (7–10). The Cys60 and Cys93 are critical amino acids for the catalytic activity of thiol peroxidase (Tpx), which can reduce H_2_O_2_, cumene hydroperoxide, and t-butyl hydroperoxide (11). The Cys61 and Cys174 of AhpC as well as Cys133 and Cys130 of AhpD were confirmed to be critical for the activity of AhpC and AhpD (12). Besides, the reversible oxidations of certain protein thiol groups play key signal roles in a series of physiological processes and defense against oxidative damages. Some of these cysteine modifications can be reversed by the thioredoxin and MSH system through a reduction process (7, 9). Due to the reversibility and transientness of cysteine redox state changes and the relatively low cysteine content in the entire proteome, detecting and quantifying these cysteine modifications are challenging. Therefore, identifying and quantifying proteins and cysteine residues affected by oxidative stress in *Mtb* will provide us with a deeper understanding of the oxidative stress response and new inspiration for the screening and design of anti-TB drugs.

SOH is one of the oxidation forms of cysteine sulfhydryl, which plays a significant role in redox regulation (4). The main inducer of SOH is H_2_O_2_, which is the product of the catalytic activity of superoxide dismutase. SOH can modify the activity of enzymes and can be the initiator of disulfide bond formation, which is also an indicator of severe oxidative damage (13). In order to effectively identify and quantify SOH, the methods for selectively labeling and enriching need to be developed first. In the past few years, proteomics based on LC-MS/MS has been widely used in the identification of phosphorylation, glycosylation, acetylation modifications, etc. (14). In addition, the development of sulfhydryl activity probes for labeling cysteine residues and proteomics methods for isolating and identifying proteins with modifications have made it possible to conduct cysteine-related research (15, 16). However, many thiol modifications are relatively unstable, and the thiol itself is easily modified artificially during protein separation and labeling. Therefore, to reliably identify and quantify protein sulfhydryl groups in biological samples, the prerequisite is to effectively capture sulfhydryl groups in the natural redox state. To explore the cysteine modification changes of *Mtb* H37Rv under oxidative stress, in our study, H_2_O_2_ was used to provide the oxidative stress, reactive sulfhydryl alkylating reagents such as N-ethylmaleimide (NEM) or S-methylthiomethanesulfonate (MMTS) were used to block all free sulfhydryl groups, and then SOH modifications were selectively reduced and labeled. Besides, a cysteine-specific iodoacetyl equivalent tandem mass tag (IodoTMT) kit containing a reactive group, a reporter group, and a balance group, was used for the label and quantification of cysteine modifications (17, 18). Total cysteine (TC) modification and SOH modifications peptides and proteins were detected separately, and related proteins with significant changes were identified and quantified. The result provided a firsthand view of the cysteine-modified proteome of *Mtb* under oxidative stress.

## RESULTS

### Identification of peptides and proteins with cysteine modification.

**FIG 1:**
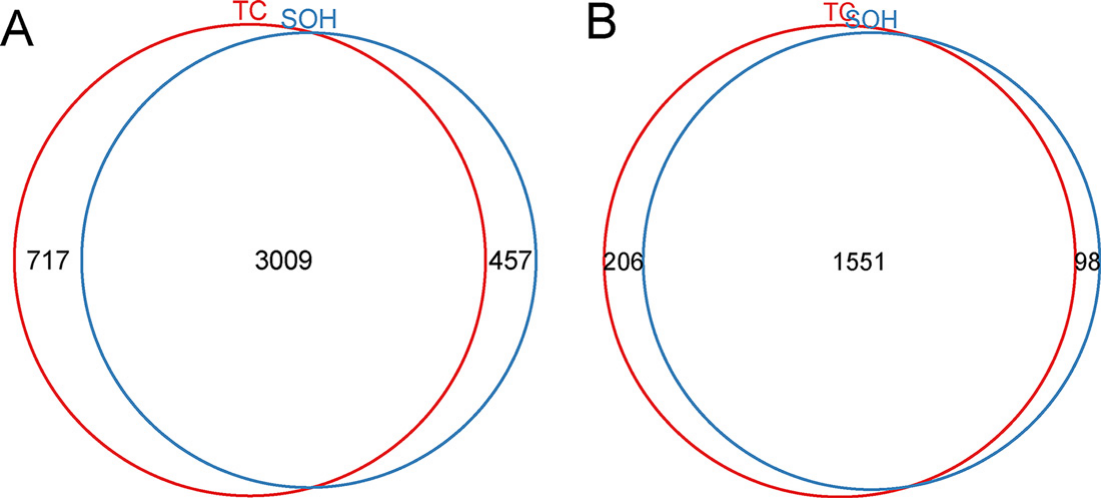
The Venn plot of modified peptides and proteins identified in *Mtb* between TC and SOH under H_2_O_2_ pressure. A, for labeled peptides. B, for labeled proteins. TC, total cysteine modification; SOH, S-sulfenylation modification.

IodoTMT6plex reagents have been used in the study to determine proteins with cysteine modification sites (19). In our study, for TC modification analysis, 7,991 peptides were identified, among which 3735 were peptides with the cysteine residue, and 3,726 cysteine-contained peptides representing 1,750 proteins that were successfully labeled with iodoTMT. For SOH modification analysis, 8,054 peptides were identified, among which 3,504 were peptides with the cysteine residue, and 3,466 cysteine contained peptides representing 1,650 proteins that were successfully labeled with iodoTMT. To compare the difference of modified peptides and proteins between TC and SOH, the Venn plot is shown in Fig. 1. There were 3,009 labeled peptides of the two groups that overlapped; 717 peptides were only in TC, and 457 peptides were only in SOH (Fig. 1A). There were 1,551 labeled proteins of the two groups that overlapped; 206 were only in TC, and 98 were only in SOH as shown in Fig. 1B.

In the study, H_2_O_2_ was used to provide the oxidative stress, and peptides and proteins changes of TC and SOH were studied separately by IodoTMT6plex quantitative proteomics. As showing in Fig. S1 and 2, all the replicates showed linear correlations in both groups. For TC analysis, after proteins were filtered by protein false discovery rate(FDR) < 0.01, proteins with missing values were excluded. Finally, 1,664 proteins were scaled for statistical analysis, and 279 proteins were filtered by *P* < 0.05 (Table S1). For SOH analysis, 1560 proteins were scaled for statistical analysis, and 296 proteins were filtered by *P* < 0.05 (Table S2). To compare the difference of significantly modified proteins between TC and SOH groups under H_2_O_2_ pressure, the Venn plot was drawn as shown in Fig. S3, and 68 proteins overlapped in TC and SOH groups, while 211 were only in TC, and 228 were only in SOH. When comparing the numbers of modified sites in the 68 overlapped proteins (Table S3), we found that the modified numbers of most proteins in the SOH group were less than or equal to those in the TC group. In the meantime, a certain proportion of carbamidomethyl modification was observed in the SOH-modified sites while only total cysteine modification was observed in the TC group.

### Identification of proteins with cysteine modification under oxidative stress.

Among the 279 proteins with significantly different levels (*P* < 0.05) of cysteine modification under H_2_O_2_ pressure, the modification levels of 58 proteins were significantly changed filtered by fold change >1.2 and *P* < 0.05 (Table S4). As shown in Fig. S4A, the TC modification levels of 28 proteins were increased while those of 30 proteins were decreased. Proteins related to zinc ion binding, oxidation-reduction process, and metal ion binding were most enriched according to the GO results shown in Fig. S4B. And most significantly changed proteins were involved in metabolic pathways, biosynthesis of secondary metabolites, and microbial metabolism in diverse environment pathways according to the KEGG pathway enrichment results (Fig. S5).

### Identification of proteins with SOH modification under oxidative stress.

**FIG 2:**
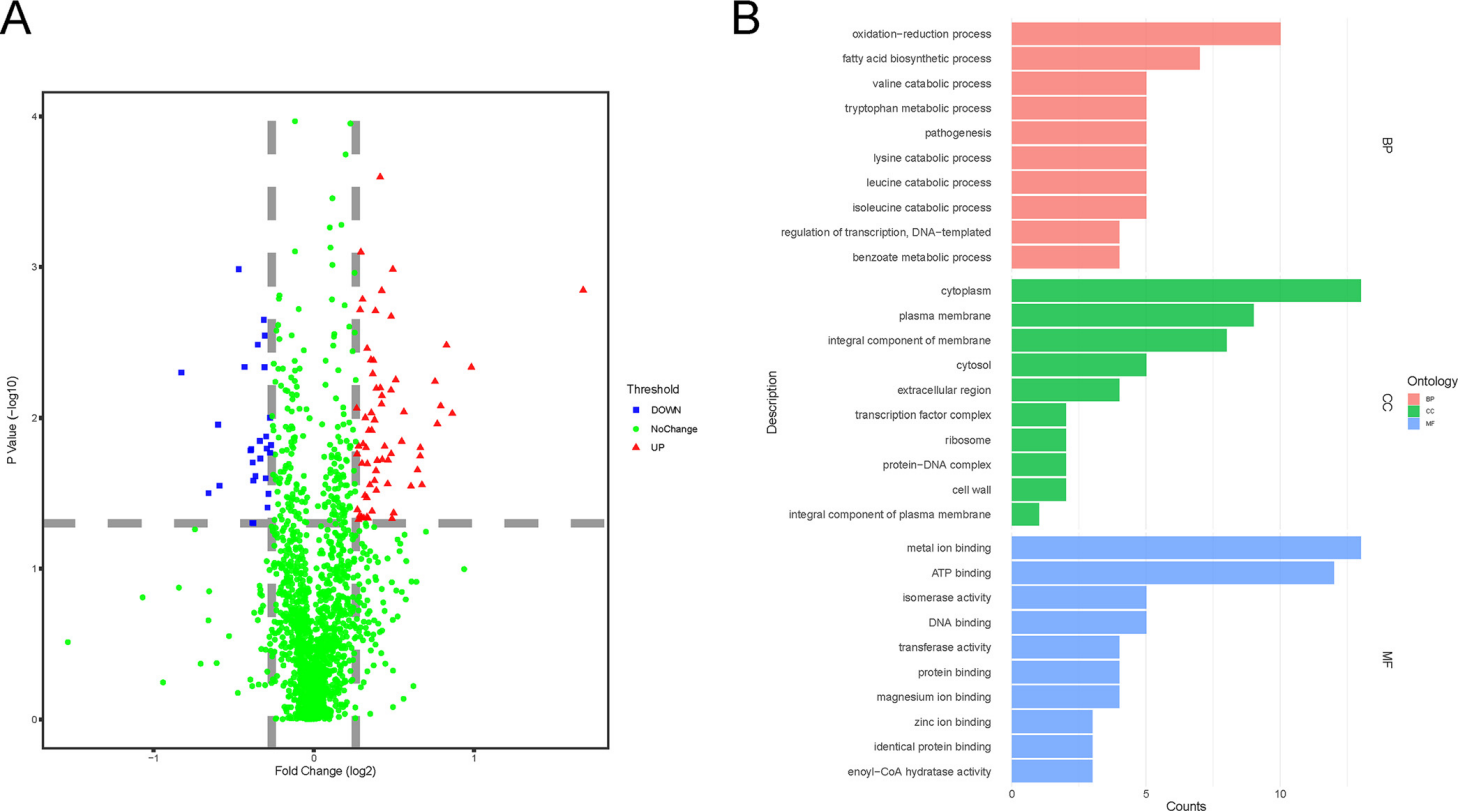
A The volcano plot of *Mtb* SOH-modified proteins under H_2_O_2_ pressure; B, GO enrichment results of SOH-modified proteins with significantly changed levels.

Among the 296 proteins with significantly different levels (*P* < 0.05) of SOH modifications under H_2_O_2_ pressure, most proteins contained 1 to 7 modified sites, which accounted for 97% of the total SOH modified proteins as shown in Fig. S6. P9WQE7, P9WGY9, and O06560 have 10 modified sites, while O50431, A0A089QRB9, O53669, P9WIV9, and I6Y231 have 9, 11, 12, 13, and 14 modified sites. Among these proteins, the SOH modification levels of 89 proteins were significantly changed filtered by fold change >1.2 and *P* < 0.05 (Fig. 2A). After normalization by TC modification ratios in Table S3, the final list of proteins with significantly changed SOH modification levels was shown in Table S5. The modification levels of 22 proteins were decreased, while those of 67 proteins were increased.

**FIG 3:**
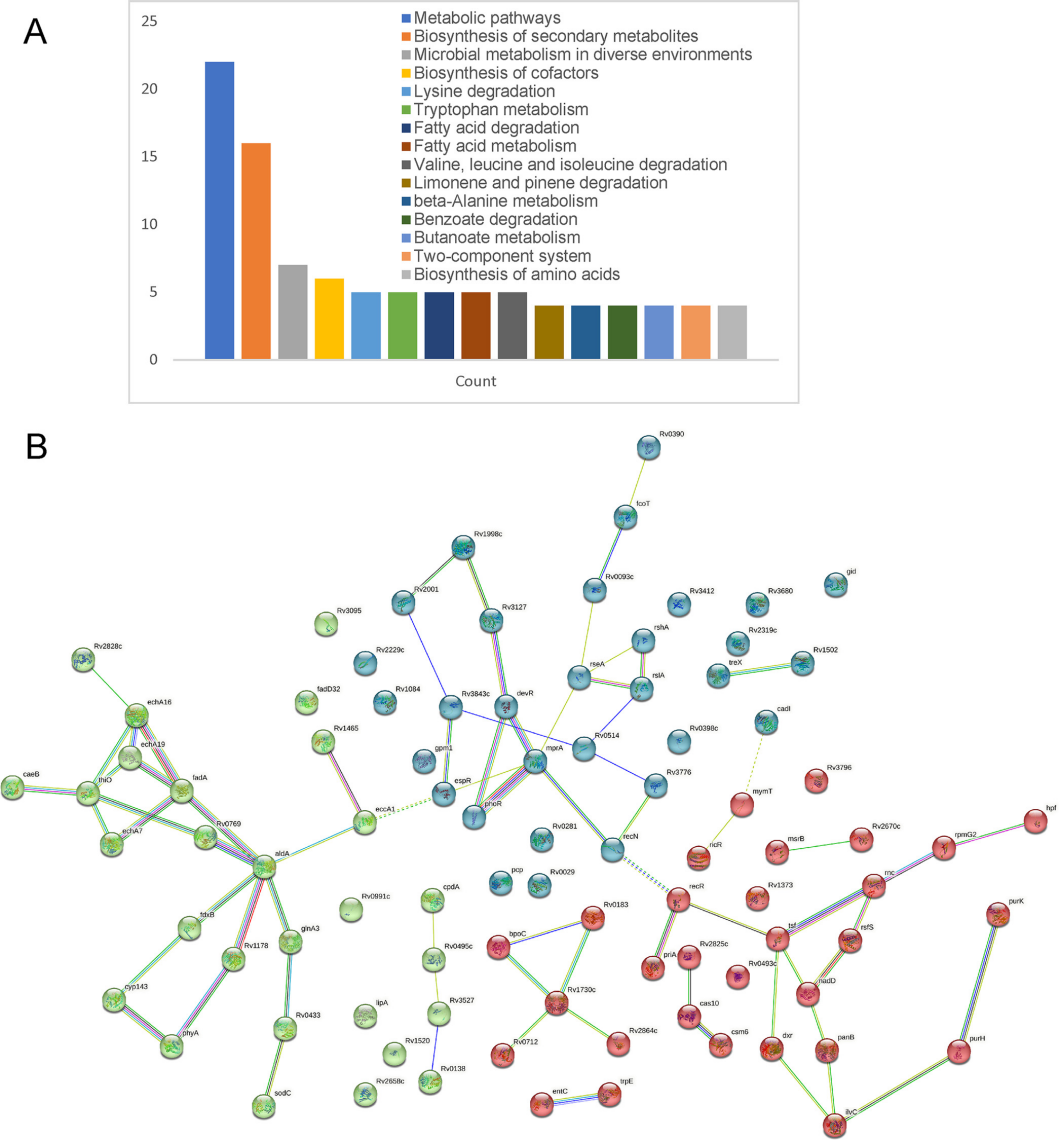
A KEGG pathway enrichment results of proteins with significantly changed SOH-modified levels; B, STRING analysis results of proteins with significantly changed SOH-modified levels.

These proteins are located in the cytoplasm (15), cell membrane (4), secreted (3), and unknown (others) according to subcellular location present in the Uniprot database. And six of them have transmembrane parts. According to the GO enrichment results shown in Fig. 2B, most of these proteins were related to the oxidation-reduction process, fatty acid biosynthetic process, etc. in BP; cytoplasm, plasma membrane, integral component of membrane, etc. in CC; and metal ion binding, ATP binding, DNA binding, etc. in MF. According to the KEGG pathway enrichment results shown in Fig. 3A, most of the proteins were involved in the metabolic pathways, biosynthesis of secondary metabolites, microbial metabolism in diverse environments, etc. Interestingly, the modification levels of FadA (*Rv0859*), EchA7 (*Rv0971c*), EchA16 (*Rv2831*), and EchA19 (*Rv3516*) were all increased, which were involved in fatty acid metabolism, valine, leucine and isoleucine degradation, lysine degradation, fatty acid degradation, tryptophan metabolism, benzoate degradation, butanoate metabolism pathways, etc. The SOH modification levels of AldA (*Rv0768*), which is involved in 18 pathways, were decreased. And clusters of genes that have strong interactions with each other were observed in the STRING analysis results (Fig. 3B), and genes such as *aldA*, *echA7*, *fadA*, *echA16*, *thiO*, etc., constituted the hub of the interaction network, indicating the vital roles played under oxidative stress.

### Proteins with significantly changed SOH-modified levels under oxidative stress.

Among the 89 SOH-modified proteins which were identified as significantly altered levels of SOH modifications under H_2_O_2_, clusters were observed according to the STRING result shown in Fig. 3B. Acyltransferase FadA (O53871, *Rv0859*), enoyl-CoA hydratase EchA7 (P71540, *Rv0971c*), enoyl-CoA hydratase EchA16 (I6YEH6, *Rv2831*), oxidoreductase (P9WGQ9, *Rv0769*), thiamine biosynthesis oxidoreductase ThiO (P96261, *Rv0415*), stress-related carboxylesterase B CaeB (P9WHR5, *Rv2223c*), PSase PhyA (P9WHP3, *Rv3397c*), cholesterol catabolism related enoyl-CoA hydratase EchA19 (O53561, *Rv3516*), gathered together and the SOH modification levels of these proteins were all increased. Among them, FadA, was formerly reported as a hypoxia-induced mycobacterial protein suppressing host immunity via modulation of host fatty acid metabolism (20). CaeB, is a secretary, carboxyl-esterase, with enhanced expression under acidic and nutritive stress conditions and might contribute to the intracellular survival of bacteria (21). The cluster also included glutamine synthase GlnA3 (O07752, *Rv1878*), aldehyde dehydrogenase AldA(I6X9R9, *Rv0768*), FadD32 (O53580, *Rv3801c*), cytochrome P450 143 (P9WPL3, *Rv1785c*), and aminotransferase (O50434, *Rv1178*), and the SOH modification levels of these proteins were decreased, while that of FdxB (P71846, *Rv3554*) was increased. The SOH modification levels of proteins such as P9WH95 (*rpmG2*), P9WNM1(*tsf*), P9WMG3 (*mxyR*), L0T905 (*rseA*), P9WJ69 (*rshA*), P9WJ67 (*rslA*), O05886 (*hpf*), P9WH03 (*rnc*), O86327 (*rsfS*), P9WMQ9 (*priA*), P9WHI3 (*recR*), P9WHI7 (*recN*), P9WMF9 (*devR*), P9WJB7 (*espR*), and P9WGW9 (*rsmG*), which were related to transcription and translation process, were all increased, except for O86327 (*rsfS*), a ribosomal silencing factor, whose SOH modification levels decreased. Among them, L0T905 (*rseA*), P9WJ69 (*rshA*), and P9WJ67 (*rslA*) were reported to be ZAS proteins, which release their cognate σ factor in response to oxidative stress (22). What’s more, the modification levels of hibernation promoting factor HPF (O05886, *Rv3241c*), which was related to ribosome protection during dormancy (23) increased under oxidative stress. And, the SOH modification levels of stress-related proteins such as peptide-methionine (R)-S-oxide reductase MsrB (I6YA00, *msrB*), which was reported as the enzymatic defenses of *Mtb* against ROI and RNI (24), universal stress protein Rv2319c (P9WLB5, *Rv2319c*), superoxide dismutase [Cu-Zn] SodC (P9WGE9, *Rv0432*), and nonheme bromoperoxidase BpoC (P9WNH1, *Rv0554*) were all increased. SodC is a Cu, Zn superoxide dismutase which contributes to the resistance of *Mtb* against oxidative burst products generated by activated macrophages (25). SodC and BpoC were also proved to be involved in the protection from oxidative stress in a dormant, nonreplicative state (26). EccA1 (P9WPH9, *Rv3868*) is an essential CbxX/CfqX-family ATPase of the *Mtb* ESX-1 secretion system (27). EspR (P9WJB7, *Rv3849*), a regulator of the ESX-1 secretion system in *Mtb*, is directly regulated by the two-component systems MprAB and PhoPR (28). Interestingly, MprA (P9WGM9, *Rv0981*), PhoR (P71815, *Rv0758*), and DevR/DosR response regulator (P9WMF9, *Rv3133c*) showed a close relationship with each other. MprA has a vital role in the establishment and maintenance of persistent, latent infection by *Mtb*. DevR/DosR response regulator (P9WMF9, *Rv3133c*) is believed to participate in virulence, dormancy adaptation, and antibiotic tolerance mechanisms of *Mtb*. Previous studies have clarified that M. smegmatis high-level induction of DevR occurred in response to 7 mM H_2_O_2_ (29, 30). In our research, the SOH modification levels of EccA1, EspR, MprA, and DevR/DosR increased while phoR decreased. Formylglycine-generating enzyme FGE (I6Y8I5, *Rv0712*) converts a specific cysteine in newly synthesized sulfatases to formylglycine (FGly), which plays essential roles in development and homeostasis (31). MymT (P9WK09, *Rv0186A*), a copper-protective metallothionein, has four cysteine residues in the 53 amino acid sequence. Interestingly, the SOH modification levels of MymT increased highest among these proteins. And the increased modification levels of MymT promoted the modification levels of the copper-sensing transcriptional repressor RicR (O07434, *Rv0190*).

As shown in Fig. 3B, bifunctional purine biosynthesis protein PurH (P9WHM7, *Rv0957*) with increased SOH modification levels have strong interaction with PurK (P9WHL9, *Rv3276c*), and IlvC (P9WKJ7, *Rv3001c*) with decreased SOH modification levels. Interestingly, the modified long-chain fatty acyl-CoA thioesterase FcoT (P9WM67, *Rv0098*), which is essential for *Mtb*'s survival in mouse macrophages (32), and DXP reductoisomerase Dxr (P9WNS1, *Rv2870c*), an essential gene of *Mtb* (33), were also accumulated under oxidative stress. Furthermore, the SOH modification levels of cell wall synthesis related glycolipid sulfotransferase (P9WGB9, *Rv1373*), penicillin-binding protein P71988 (*Rv1730c*), and penicillin-binding lipoprotein O33346 (*Rv2864c*) were all increased. The SOH modification Cas10 (P71629, *Rv2823c*), and Csm6 (P71635, *Rv2818c*) belonging to CRISPR system were also identified. And the modification levels of O53156 (*Rv1465*), which is the part of suf operon encoding the primary Fe-S cluster biogenesis system, decreased.

## DISCUSSION

The ability to maintain RH is vital for *Mtb* to survive the host environment even under drug treatment pressure. One study has reported that auranofin was active against Gram-positive bacteria by inhibiting the bacterial thioredoxin reductase, a protein essential in many Gram-positive bacteria for maintaining the RH balance and protecting against oxidative stress (34). Thus, to discover drug targets based on the proteins related to RH is promising and meaningful for anti-TB treatment. In our study, oxidative stress was provided by H_2_O_2_ *in vitro*, and proteins and peptides with cysteine residue that were influenced were labeled by iodoTMT reagents and quantified. These data provided us with a brand new map of the proteins with cysteine modifications involved in the RH, along with new hints for anti-TB drug target discovery.

According to the identification results, 47% of identified peptides contained cysteine residues, among which 99% were labeled with IodoTMT for TC analysis. 44% of identified peptides contained cysteine residues, among which 99% were labeled with IodoTMT for SOH analysis. Labeled efficiency was high, but more than half of the identified peptides were unlabeled peptides, indicating the limitation in the purification and enrichment progress, though anti-TMT enrichment was used. Venn plot results of proteins and peptides identified suggested that most of them were overlapped, indicating that more than 80% cysteine-contained peptides could be modified as SOH. Interestingly, when comparing the significantly differentially expressed proteins acquired from TC and SOH analysis (Fig. S3), there were only 68 that overlapped, with 211 in TC and 229 in SOH. Among them, the modification levels of only four proteins were increased in both TC and SOH analysis, while those of three proteins were decreased. So, what causes this difference in the differentially modified levels under oxidative stress? The modified sites of 68 overlapped proteins were analyzed, and we found that for the cysteine sites in some proteins, only part of them were modified as SOH, while the whole sites were modified for the TC. We speculated that the reason might be multiple modification forms of the cysteine residues in some proteins were identified in the TC group.

Meaningfully, the SOH modification levels of stress-related proteins such as ThiO, RseA, RshA, RslA, MsrB, Rv2319c, SodC, BpoC, and functionally related EccA1, EspR, MprA, and DevR/DosR were all increased under oxidative stress, indicating the roles of these proteins in maintaining the RH status. According to GO enrichment results of significantly changed SOH modification levels of proteins, 10 proteins were related to the oxidation-reduction process, including AldA, MsrB, ThiO, SodC, Rv0769, IlvC, Rv3127, BpoC, Dxr, and CYP143. The SOH modification levels of all these proteins were increased, except for AldA, IlvC, and CYP143, to resist oxidative pressure. Given the difficulties of *Mtb* research, limited proteins were found related to RH. MsrB (I6YA00), Thiol peroxidase (Tpx, P9WG35), and Alkyl hydroperoxide reductase C (AhpC, P9WQB7) have been reported as the key elements in the defense against oxidative stress in *Mtb* (24, 35, 36). And the cysteine of Thiol peroxidase from Escherichia coli and MsrB from C. diphtheriae could be oxidized to sulfuric acid (SOH) by peroxide (37, 38). Interestingly, elongation factors were reported as targets of oxidation by ROS via the oxidation of cysteine residues to SOH (39). In our study, the SOH modification levels of elongation factor Ts (P9WNM1, *tsf*) were significantly increased under oxidative pressure.

The iodoTMT protocol was easy to get used to and has been applied in many cysteine modification researches. In our study, it is the first time to identify the total cysteine and SOH modification in *Mtb* proteome. However, there were some limitations. According to the results of identified peptides, lots of cysteine-free peptides were identified, indicating the enrichment method has limited specificity. Proteins like alkyl hydroperoxide reductase E (AhpE) were missed in the results, which was previously proved to react fast with hydroperoxides, forming a stable sulfenic acid(SOH) (40). In addition, the stability of the reagent is limited and the total conducted time for the entire experiment needs to be controlled. *Mtb*, a slow-growing aerobic bacillus, has a special cell wall structure. Thus, extraction and digestion of *Mtb* proteins were challenging. Moreover, the specific roles of SOH-modified sites of proteins in maintaining RH need further exploration. But it is difficult to purify and express proteins with mutant cysteine sites because of the high GC characteristics of the nucleic acid sequence. In the follow-up research, we will try to overcome these difficulties to verify the cysteine function in proteins. In addition to the limitation of existing quantification methods, investigating cysteine modification using *in vitro* model cannot completely reflect the modification conditions *in vivo*.

In summary, our study demonstrated the cysteine-related proteome responses of *Mtb* under oxidative stress, and the SOH modification levels of proteins related to the oxidation-reduction process, fatty acid biosynthetic process, stress response, protein repair, cell wall, etc., were changed. Given that the ability of *Mtb* to resist oxidative stress is extremely important for maintaining RH and survival in the host, discovering protein modification changes under oxidative stress is essential to elucidating its antioxidant mechanism. Our results provided a list of proteins involved in the antioxidant process that potentially could be considered targets for drug discovery and vaccine development. Furthermore, it is the first study to determine and quantify the SOH-modified proteins in *Mtb*, which provided better insight into the *Mtb* response to the host oxidative defense and enable a deeper understanding of *Mtb* survival strategies.

## MATERIALS AND METHODS

### Cultivation of *Mtb* and *in vivo* oxidation.

*Mtb* H37Rv was cultivated in 7H9 medium supplemented with 0.2% (vol/vol) glycerol, 0.05% (vol/vol) Tween 80, and 10% (vol/vol) oleic acid albumin dextrose catalase (OADC, BD Company, USA) at 37°C until OD_570_ reached 0.7. Then 50 mM H_2_O_2_ was added in the experimental group for 30 min, three biological replicates were conducted in each group. Cells were centrifuged at 5 000 × *g* for 10 min and washed twice in PBS (pH 7.4). The bacteria were subsequently resuspended by lysis and blocking buffer (150 mM HEPES, pH 7.3, 50 mM MMTS, 2% SDS, 1 mM EDTA, 0.1 mM neocuproine) and sonicated (3s on, 6s rest, 30%) for lysis. Cell lysates were then precipitated with 6 volumes of ice-cold acetone for 1h at −20°C and centrifuged (10 000 × *g*, 10 min, 4°C). Then protein pellets were washed with ice-cold acetone and resuspended in AENS buffer (50 mM ammonium bicarbonate, pH 8.0, 1 mM EDTA, 0.1 mM neocuproine, 2% SDS) as previously reported (13). Protein concentration was measured using the BCA assay.

### IodoTMT labeling of TC and SOH-proteins.

IodoTMT 6plex reagents were used for the labeling of both control and stressed samples. As previously reported (13), for TC modification analysis, 100 μg of protein extracts (1 μg·μL^−1^) was reduced with 10 mM TCEP for 1 h at 50°C for TC labeling, and TCEP was then removed by 0.5 mL Zeba Spin desalting columns. Three replicate samples of the control group were labeled with 5 mM iodoTMT-126, iodoTMT-127, and iodoTMT-128, while replicate samples of experimental groups were labeled with 5 mM iodoTMT-129, iodoTMT-130, and iodoTMT-131. For SOH analysis, 1 mg of each sample (2 μg · L^−1^) was reduced with 10 mM sodium arsenite for SOH and labeled by 0.9 mM iodoTMT-126, iodoTMT-127, and iodoTMT-128 (control group), while samples of experimental groups were labeled by 0.9 mM iodoTMT-129, iodoTMT-130, and iodoTMT-131 (experimental group). The same replicate samples were used for TC and SOH analysis, and the follow-up treatment of the two groups was carried out at the same time. For all the samples, labeling reactions proceeded for 2 h at 37°C protected from light. Then the reactions were quenched by the addition of 500 mM dithiothreitol (DTT) and 15 min incubation protect from light. Equal amounts of samples of different tags were mixed. Then the mixtures were precipitated with 6 volumes of ice-cold acetone overnight at −20°C. The samples were centrifuged at 10 000 × *g* for 10 min at 4°C. Pellets were then washed with ice-cold acetone/methanol (50% vol/vol) to remove excess iodoTMT reagents and dried for 10 min.

### Peptides acquisition by enzymatic digestion and anti-TMT enrichment.

Protein pellets were resuspended in 50 mM ammonium bicarbonate and reduced with 20 mM DTT and alkylated with 55 mM iodoacetamide. Then digestion was performed by trypsin (1:50) at 37°C overnight. HLB cartridge (Oasis, Waters, USA) was used for desalting. Anti-TMT enrichment was conducted by incubation with anti-TMT resin for 2 h at RT with end-over-end shaking. Then the resin was washed by 2 M urea in TBS (2 column volumes) followed by 0.1% SDC in TBS and TBS only (3 column volumes each). Labeled peptides were eluted from the resin with 4 column volumes of TMT elution buffer. Samples after anti-TMT enrichment were desalted by C18 reverse-phase tips (Reprosil-Pur Basic C18, 5 mm, Maisch GmbH) as previously described (41, 42).

### Nano LC-MS/MS analysis and identification of cysteine oxidation sites.

The enriched labeled peptides were analyzed by Nano LC-MS/MS through a Thermo Scientific Orbitrap Fusion Lumos platform coupled with an EASY-nLC 1200. A trap column (Reprosil-Pur Basic C18 (3 mm, Maisch GmbH, Germany), 20 × 0.15 mm) and C18 column (Reprosil-Pur Basic C18 [1.9 mm, Maisch GmbH, Germany] 160 × 0.15 mm) were used for separation. After the sample was loaded on the columns, the gradient started from 10% of buffer B (80% acetonitrile with 0.1% formic acid) and then from 10% to 13% of buffer B for 6 min. Then, the gradient raised from 13% to 29% of buffer B for 78 min and from 29% to 37% for 22 min. Ultimately, the gradient ascended to 95% of buffer B and was kept for 14 min. MS/MS parameters were set as previously reported: MS1 spectrum (Orbitrap resolution, 120 000; mass range, 350~1550 *m/z*; RF lens, 50%; maximum injection time, 50 ms) and an MS2 analysis with HCD accompanied by the following conditions: Orbitrap solution, 15 000; and isolation window, 0.7 Da; collision energy, 38%; maximum injection time, Dynamic. Raw data acquired were searched against *Mtb* H37Rv database from UniProt by the Thermo Scientific Proteome Discoverer version 2.2 software. Two missed cleavages of trypsin were allowed for each peptide. Reporter abundance quantification was based on the intensity, and S/N was set as 1. Peptides with dynamic modifications of oxidation, iodoTMT6plex, and carbamidomethyl were monitored. Data were normalized by the total peptide amount and Student's *t* test was used for the hypothesis test. Peptides with FDR <0.01 were taken for further analysis. All data have been deposited to the ProteomeXchange Consortium via the PRIDE (43) partner repository with the data set identifier PXD035802.

### Bioinformatics analysis.

Venn plots were conducted by the Wu kong platform (https://www.omicsolution.com/wkomics/main/) based on R Programming Language. Peptides and proteins with significant changes were selected by *P* < 0.5 and fold change >1.2. Gene Ontology and KEGG pathway enrichment were used for the function annotations and classification of proteins. Detailed information of proteins was obtained from Uniprot (https://www.uniprot.org/). Interactions between proteins were analyzed by STRING.

### Data availability.

The mass spectrometry proteomics data have been deposited to the ProteomeXchange Consortium via the PRIDE partner repository with the data set identifier PXD035802.
