# Supplementary material for: Identification and Quantification of S-Sulfenylation Proteome of Mycobacterium tuberculosis under Oxidative Stress
Source: Microbiol Spectr. 2023 Mar 21;11(2):e03386-22. doi: 10.1128/spectrum.03386-22 (PMC10101118; doi:10.1128/spectrum.03386-22)
Supplement: Supplemental file 1 — Fig. S1 to S6. Download spectrum.03386-22-s0001.pdf, PDF file, 0.4 MB [file spectrum.03386-22-s0001.pdf]

Identification and Quantification of S-sulfenylation Proteome of *Mycobacterium tuberculosis* under Oxidative Stress

Yun Lu [a], Hongtong Chen [a], Penghe Wang [a], Jing Pang [a], Xi Lu [a], Guoqing Li [a], Xinxin Hu [a], Xiukun Wang [a], Xinyi Yang [a], Congran Li [a], Yu Lu*[b], Xuefu You*[a]

[a] Beijing Key Laboratory of Antimicrobial Agents, Institute of Medicinal Biotechnology, Chinese Academy of Medical Sciences and Peking Union Medical College, Beijing 100050, China

[b] Beijing Key Laboratory of Drug Resistance Tuberculosis Research, Beijing Tuberculosis and Thoracic Tumor Research Institute, and Beijing Chest Hospital, Capital Medical University, Beijing, China

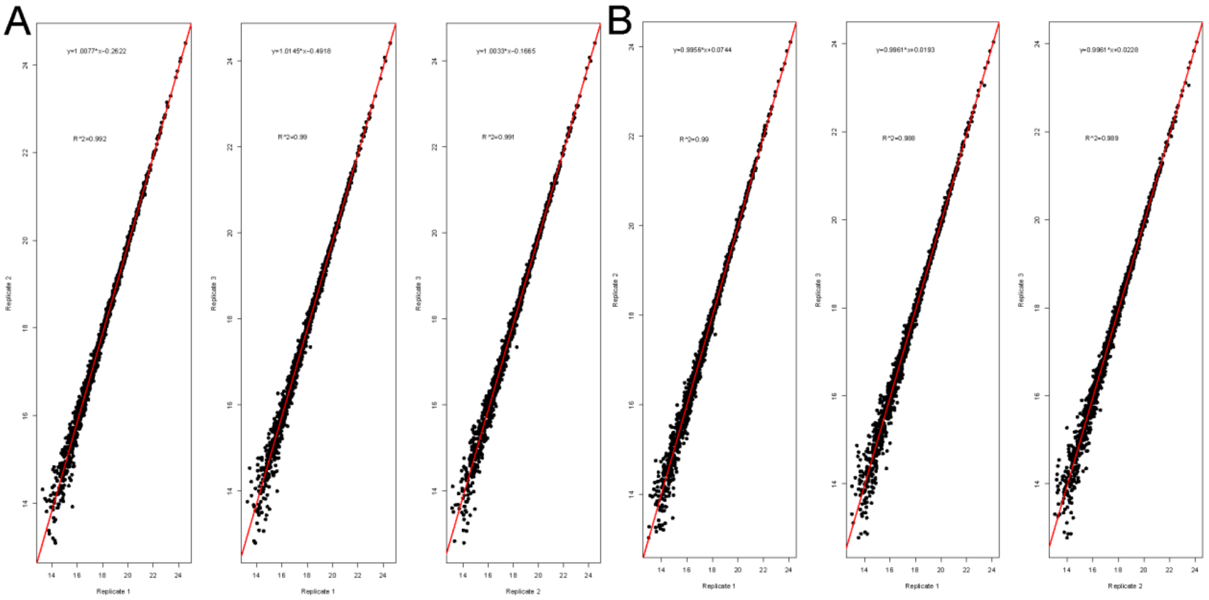

FIG S1 Linear correlation curves of replicates in control and stressed group of total cysteine modification. A, control group; B, hydrogen peroxide treated group.

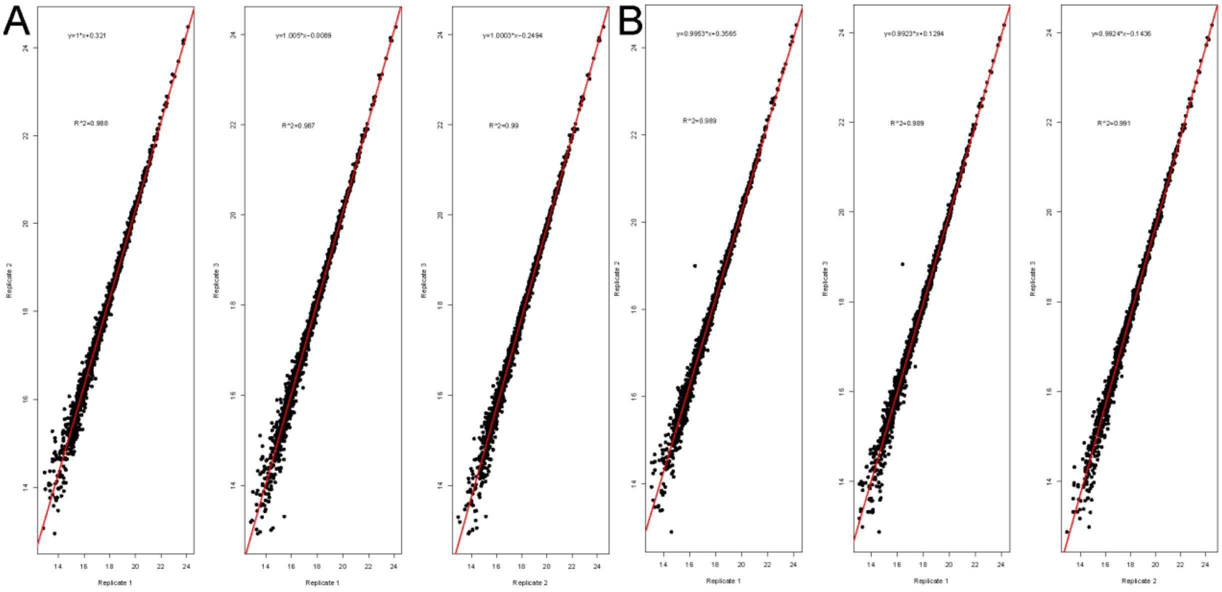

FIG S2 Linear correlation curves of replicates in control and stressed group of SOH modification. A, control group; B, hydrogen peroxide treated group.

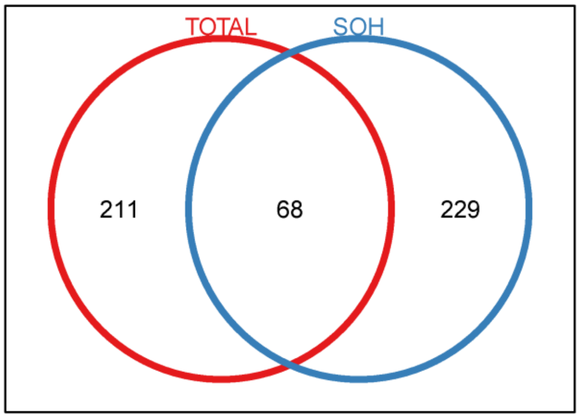

FIG S3 The venn plot of significant modified proteins ($p<0.05$) between TC and SOH groups under H_2O_2 pressure.

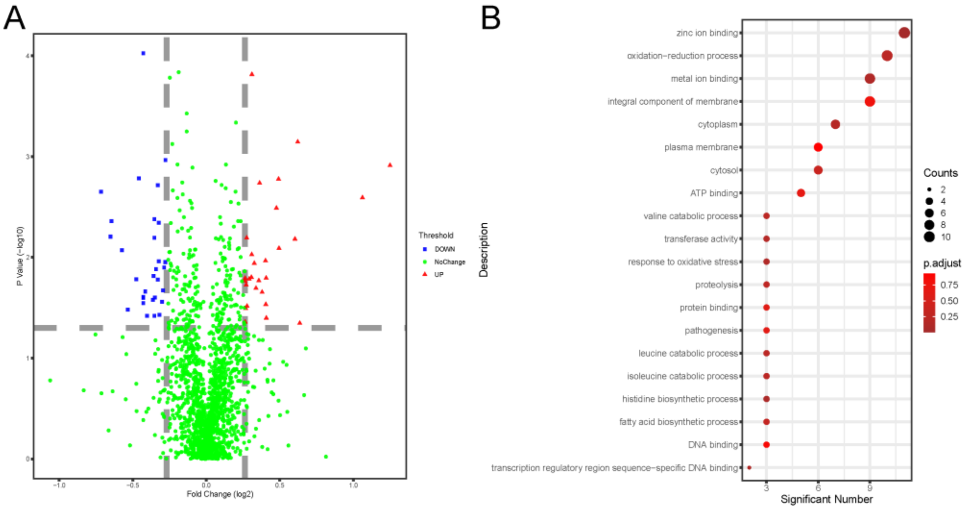

FIG S4 A Volcano plot of total cysteine modified proteins under H_2O_2 pressure; **B** GO enrichment results of proteins with significantly changed total cysteine modified levels filtered by fold change >1.2 and p <0.05. Significant number means the counts of proteins.

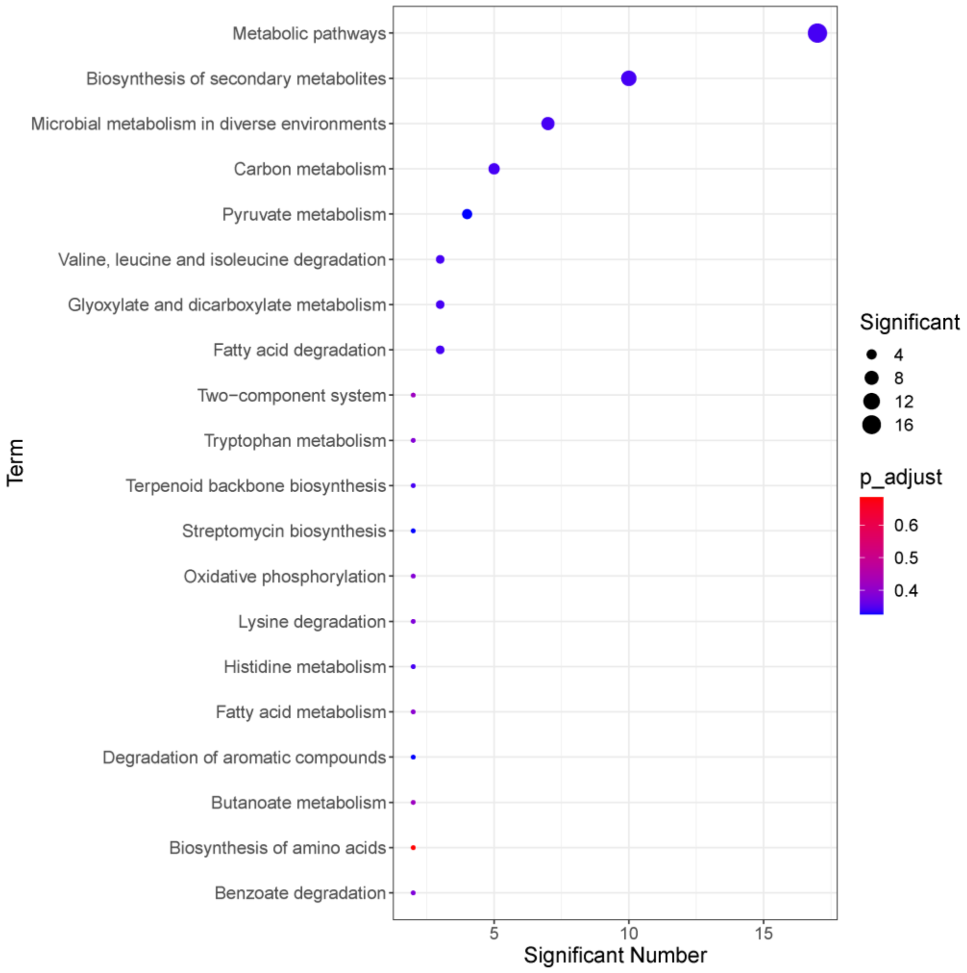

FIG S5 KEGG pathway enrichment results of proteins with significantly changed total cysteine modified levels filtered by fold change >1.2 and p <0.05.

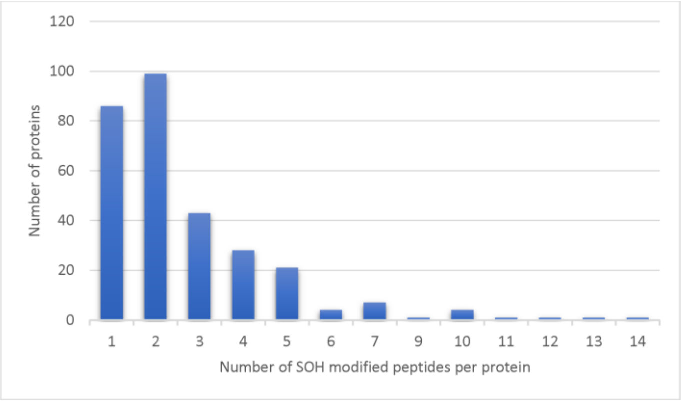

FIG S6 Number of SOH modified peptides per protein.
